# Cardiopulmonary Response to Exercise in COPD and Overweight Patients: Relationship between Unloaded Cycling and Maximal Oxygen Uptake Profiles

**DOI:** 10.1155/2015/378469

**Published:** 2015-03-19

**Authors:** Abdoulaye Ba, Fabienne Brégeon, Stéphane Delliaux, Fallou Cissé, Abdoulaye Samb, Yves Jammes

**Affiliations:** ^1^Service des Explorations Fonctionnelles Respiratoires, Hopital Nord (Assistance Publique-Hôpitaux de Marseille) and UMR MD2, Faculté de Médecine Secteur Nord, Boulevard Pierre Dramard, 13916 Cedex 20 Marseille, France; ^2^Laboratory of Physiology, Faculty of Medicine, University of Cheikh Anta Diop, Dakar, P.O. Box 45698, Dakar Fann, Dakar, Senegal; ^3^Unité Mixte Internationale Environnement, Santé, Sociétés (UMI3189 ESS), Université Cheikh Anta Diop (UCAD), P.O. Box 5005, Dakar Fann, Senegal

## Abstract

Cardiopulmonary response to unloaded cycling may be related to higher workloads. This was assessed in male subjects: 18 healthy sedentary subjects (controls), 14 hypoxemic patients with chronic obstructive pulmonary disease (COPD), and 31 overweight individuals (twelve were hypoxemic). They underwent an incremental exercise up to the maximal oxygen uptake (VO_2_max), preceded by a 2 min unloaded cycling period. Oxygen uptake (VO_2_), heart rate (HR), minute ventilation (VE), and respiratory frequency (fR) were averaged every 10 s. At the end of unloaded cycling period, HR increase was significantly accentuated in COPD and hypoxemic overweight subjects (resp., +14 ± 2 and +13 ± 1.5 min^−1^, compared to +7.5 ± 1.5 min^−1^ in normoxemic overweight subjects and +8 ± 1.8 min^−1^ in controls). The fR increase was accentuated in all overweight subjects (hypoxemic: +4.5 ± 0.8; normoxemic: +3.9 ± 0.7 min^−1^) compared to controls (+2.5 ± 0.8 min^−1^) and COPDs (+2.0 ± 0.7 min^−1^). The plateau VE increase during unloaded cycling was positively correlated with VE values measured at the ventilatory threshold and VO_2_max. Measurement of ventilation during unloaded cycling may serve to predict the ventilatory performance of COPD patients and overweight subjects during an exercise rehabilitation program.

## 1. Introduction

Predictive values of tests exploring mild physical activity have been already reported. Thus, a strong association was reported between the 6 min walk distance (6 MWD) and peak oxygen uptake (VO_2_max) in patients with cardiac and pulmonary disease [1, 2]. Unloaded pedalling represents a reproducible warm-up exercise bout. It can be taken by subjects with low to moderate exercise capacity and allows simultaneously measuring ventilation and oxygen uptake (VO_2_). We hypothesized that this mild exercise test may serve to predict the cardiopulmonary responses to heavy exercise.

Cardiopulmonary limitation at peak exercise is well documented in patients with COPD [3–5] and in individuals with overweight [6–10]. Both subjects have risk of pulmonary hyperinflation due to expiratory flow limitation. Moreover, severe overweight often results in hypoxemia. On the other hand, we found no data in the literature on the response of COPD and overweight subjects to unloaded cycling.

Chronic hypoxemia present in patients with COPD and also in numerous subjects with severe overweight may modify their response to unloaded cycling. Indeed, we already showed in healthy humans that experimental normobaric hypoxemia attenuated the ventilatory but not the cardiac response to unloaded cycling [11]. Mimicking overweight condition in healthy subjects also reduced their ventilatory response to submaximal exercise [12, 13]. Based on these data obtained in situations mimicking hypoxemia or overweight in healthy subjects, we hypothesized that the response to unloaded exercise may differ between patients with hypoxemia or overweight only. This might affect the supposed predictive values of their cardiopulmonary responses to unloaded pedalling.

## 2. Methods 

### 2.1. Patients

The study population consisted of 63 age-matched male subjects (18 normal sedentary subjects; 14 normal-weight hypoxemic patients with COPD; and 31 individuals with overweight; among them 12 were hypoxemic). Their physical characteristics are shown in Table 1. Control subjects had no medical illness at the time of the study, none were smokers, and none were involved in an exercise training program. COPD was diagnosed according to the criteria of the ATS/ERS task force [14]. The patients had not experienced any respiratory tract infection or any exacerbation of their disease for at least 4 wk before the study and received no oral corticosteroid therapy but all were treated by inhaled corticosteroids (360 *μ*g/day) and cholinergic antagonist (tiotropium bromide, 22.5 *μ*g/day). There was no supplemental oxygen during the week before the protocol. Patients with overweight were not hypertensive and did not receive any medication which could interact with the exercise response. Procedures were carried out with the adequate understanding and written consent of the subjects who all signed the informed consent form. The protocol was approved by our institutional ethics committee.

### 2.2. Study Design

The subject performed an exercise on an electrically braked cycloergometer (Ergometrics ER 800, Jaeger, Germany) connected to a microcomputer software. The testing protocol consisted in a 5 min rest period followed by a 2 min zero-watt work load period at 1 Hz cycling frequency (unloaded pedaling). Then, the load was increased as a ramp of 20 watt/minute (W/min) until the predicted maximal VO_2_ was reached (healthy subjects) or when the patient decided to interrupt the exercise bout (symptom-limited VO_2_max). Instantaneous heart rate (HR) and breath-by-breath ventilatory data were averaged every 10 s. Because the entrainment of the breathing frequency by exercise rhythm has been documented [15, 16], we imposed a constant pedaling rate of 60 revolutions per minute (rpm) which was maintained by all subjects throughout all the exercise challenge.

### 2.3. Methods

All subjects had measurements of forced vital capacity (FVC), forced expiratory volume in one second (FEV1), and total lung capacity (TLC) with a whole body pressure displacement plethysmograph (MasterLab Jaeger, Bunnik, The Netherlands). Reference values were those proposed by Quanjer [17]. Prior to the experiment, the ear lobe was pretreated with a vasodilator cream. Then, it was incised to sample arterialized blood in 100 *μ*L heparinized capillary tubes. Oxygen (PaO_2_) and carbon dioxide (PaCO_2_) partial pressures and arterial pH (pHa) were measured (Corning-Chiron model 860, Bayer Corporation, East Walpole, MA, USA). Predicted PaO_2_ value took into consideration the corresponding PaCO_2_ level, pHa, and total hemoglobin [18]. At rest, then at determination of the ventilatory threshold (VTh) and VO_2_max, blood gas tensions were analyzed. The percutaneous oxygen saturation (SpO_2_, %) was continuously measured with a pulse oximeter whose accuracy equals plus or minus two oxygen saturation percentage points between saturations of 70–100% (NPB 40, Nelcor Puritan Bennett, Pleasanton, CA, USA).

The subject wore a face mask forming an air-tight seal over the nose and mouth. Ventilation was measured with a volumetric rotor transducer (Triple V digital volume transducer, Jaeger, Germany). A side port was connected to fast-response differential paramagnetic O_2_ and CO_2_ analyzers (Jaeger: 90% response time in 100 ms). The software (Oxycon Beta, Jaeger, Germany) computed breath-by-breath data of minute ventilation (VE), respiratory frequency (fR), tidal volume (VT), oxygen uptake (VO_2_) and carbon dioxide production (VCO_2_), and the ventilatory equivalents for O_2_ (VE/VO_2_). The ventilatory threshold (VTh) corresponded to the VE and VO_2_ values at which the ventilatory equivalents for O_2_ (VE/VO_2_) exhibited a systematic increase without a concomitant increase in VE/VCO_2_ [19]. The heart rate was continuously monitored (Cardiognost Hellige, Stuttgart, Germany) and simultaneously stored with ventilatory data on the computer.

### 2.4. Data Analyses

Data are presented as mean ± standard error of mean (SEM). Statistical inferences were made by the two-way analysis of variance (ANOVA) for repeated measures at predetermined epochs (rest, 10 s, 30 s, 60 s, and 120 s of unloaded cycling), patients with COPD or overweight subjects versus controls being one factor and time course being the second factor. Comparisons between groups or over time were performed taking into account the difference between each variable measured at a time during unloaded exercise and its baseline value. Correlations between variables were evaluated with Spearman's test. Cardiac and respiratory variables obtained at rest were compared using a stepwise multiple linear regression model to determine an equation that would best predict the cardiopulmonary response during unloaded cycling. The same statistical analysis allowed linking plateau values of variables measured during unloaded cycling with those measured at VTh and VO_2_max. The potential coefficients of equation included HR, fR, VT, VE, VO_2_, and PaO_2_. Significance was set at the 0.05 level.

## 3. Results

### 3.1. Baseline Variables

Table 1 shows that subjects with overweight had significant lower total lung capacity (TLC) and expiratory reserve volume (ERV) compared to COPDs and normal-weight normoxemic controls. According to the ATS/ERS task force [14], our COPD patients had moderate airway obstruction (FEV1/FVC < 70%; 50 < FEV1 < 80% predicted), hypoxemia (58 < PaO_2_ < 70 mmHg; 93 < SpO_2_ < 97%), and no hypercapnia. Hypoxemic overweight subjects had the highest body mass index and the lowest TLC values. In the four groups (controls, hypoxemic COPDs, normoxemic, or hypoxemic overweight subjects), the resting values of HR, VE, fR, VT, and VO_2_ did not differ, allowing the consideration of the absolute variation of each variable during unloaded cycling.

### 3.2. Influence of Resting Levels of Physiological Variables on the Cardiopulmonary Response to Unloaded Cycling

Multiple regression analysis showed that, among the different variables, only the resting fR was negatively correlated with the plateau fR increase at 120 s of unloaded pedaling (Δ fR, min^−1^ = −0.42 ^*^ fR rest + 10.20; *r* = 0.521; *P* < 0.01). Despite the fact that ERV was significantly lower in overweight individuals compared to controls and COPDs, we found no correlation between the reduction of lung volumes and the ventilatory response (VE, VT, and fR) to unloaded cycling.

### 3.3. Response to Unloaded Cycling

**Figure 1:**
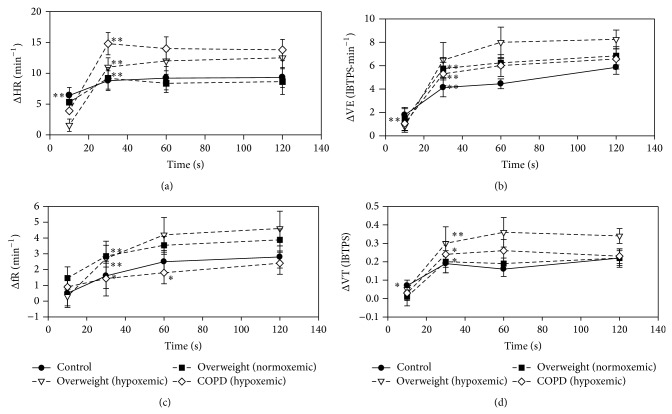
The changes in cardiorespiratory variables, related to their corresponding resting levels, during the 2 min period of unloaded cycling exercise in the four groups. Normoxemic and hypoxemic subjects are identified by black and open symbols, respectively. Up to down: heart rate (HR), minute ventilation (VE), respiratory frequency (fR), and tidal volume (VT). Values are mean ± SEM. Asterisks denote the first significant change (^*^ *P* < 0.05 and ^**^ *P* < 0.01). All further variations were significant (^**^).

At the end of the unloaded cycling period, no significant changes in resting level of SpO_2_ were measured in controls, COPDs, and overweight subjects. Table 1 reports the VO_2_ values measured at the end of the unloaded pedaling period, ventilatory threshold, and maximal workload. Two-minute unloaded cycling period increased VO_2_ value by 3.0 (overweight subjects) to 3.6 (controls and COPD), the intergroup differences being not significant.  Figure 1 shows the time course of changes in HR and ventilatory variables measured during unloaded cycling. In controls, the changes in HR, VE, and VT were already significant at 10 s and at 30 s for fR. In the other groups, the first significant increase in HR, VE, and VT was only measured at 30 s and, in COPDs, fR increase only occurred at 60 s. In the four groups, a plateau response was obtained at 120 s. HR increase was significantly accentuated in COPD and hypoxemic overweight subjects (resp., +14 ± 2 and +13 ± 1.5 min^−1^, compared to +7.5 ± 1.5 in normoxemic overweight ones and +8 ± 1.8 min^−1^ in controls). On the other hand, the fR increase was accentuated in all overweight subjects (hypoxemic: +4.5 ± 0.8; normoxemic: +3.9 ± 0.7 min^−1^) compared to +2.5 ± 0.8 in controls and +2.0 ± 0.7 min^−1^ in COPDs. We noted a gradation of the VE response to unloaded cycling in the four groups (controls: +5.6 ± 0.6 LBTPS·min^−1^; COPDs: +6 ± 0.5; normoxemic overweight subjects: +7 ± 0.4; hypoxemic overweight subjects: +8 ± 0.6).

### 3.4. Influence of the Cardiorespiratory Response to Unloaded Cycling on the Performances during Incremental Maximal Exercise

**Figure 2:**
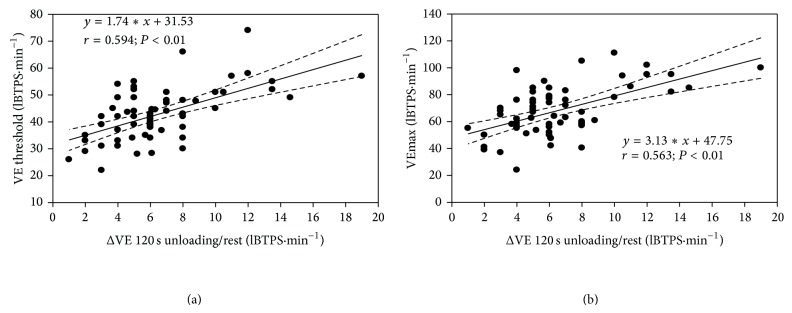
Relationships between minute ventilation measured at the ventilatory threshold (VE threshold) and maximal oxygen uptake (VEmax) during an incremental cycling exercise and the maximal VE changes measured at 120 s of unloaded cycling exercise. All subjects were pooled together for this analysis. Regression line with 95% confidence intervals.

We found no relationship between HR changes measured at the end of unloaded cycling and HR values measured at determination of the ventilatory threshold and VO_2_max. By contrast, the plateau VE increase during unloaded cycling was positively correlated with the ventilation measured at the ventilatory threshold and VO_2_max (Figure 2). No correlation was found between the plateau increases in fR and VT during unloaded cycling and their corresponding changes at the ventilatory threshold and VO_2_max.

## 4. Discussion 

The present study shows that two factors (hypoxemia and overweight) differently affect the heart rate and ventilatory responses to unloaded cycling. Thus, the plateau HR increase was higher when hypoxemia was present whereas the overweight increased the VE and fR responses. We noted that the plateau VE increase during unloaded cycling was correlated with the magnitude of hyperventilation measured at the ventilatory threshold and maximal workload. On the other hand, the plateau HR increase during unloaded cycling was not correlated to HR changes at the ventilatory threshold and VO_2_max. It was already reported that pedaling with no load before exercise did not affect the heart rate increase during incremental exercise [20]. The energy cost of the 2 min unloaded cycling period was commensurate with that measured during walking at 1 km·hr^−1^ on a treadmill [21, 22]. Thus, unloaded cycling elicited a similar response than walking at a low rate on a flat ground.

Our study has evidenced a clear relationship between the resting respiratory frequency and its plateau value during unloaded cycling, the individuals having the lowest respiratory frequency exhibiting the highest response. We already reported in healthy sedentary subjects that the ventilatory response to the activation of different respiratory afferents depends on the breathing pattern at rest. Thus, in resting [23] and exercising subjects [24], the entrainment of the breathing rhythm by high frequency mechanical muscle stimulation was only significant in individuals having the slowest respiratory frequency at rest. The same relationship was also found between the breathing response to transient hypercapnia and the resting breathing pattern [25]. Thus, the role played by respiratory afferents seems to be accentuated in subjects having a slow and ample spontaneous breathing pattern.

The present data in COPDs and also in overweight subjects with chronic hypoxemia partly confirm our previous observations in healthy subjects exposed to acute normobaric hypoxemia [11], an experimental condition which delayed the VE and HR responses to unloaded cycling and markedly lowered both the plateau increases in VE and HR. In the present study, all subjects with chronic hypoxemia had delayed VE and HR responses to unloaded cycling but the plateau HR increase was accentuated. We noted that the magnitude of plateau VE increase did not vary in hypoxemic COPDs but only increased in overweight subjects. Thus, the sole common point between the present study and the previous observations was a delayed cardiopulmonary response to unloaded cycling exercise in both acute and chronic hypoxemic conditions. The slowed kinetics of minute ventilation and heart rate changes in COPD patients and also in overweight subjects may be related to the reduction of blood and oxygen supply to limb muscles. Indeed, a limitation of oxygen uptake in the lower limbs during cycling exercise is documented in patients with COPD [26] and the peripheral vascular conductance is reduced in obesity [8]. Animal experiments have revealed that group III-IV muscle afferents, which play key role in the cardiopulmonary response to exercise via the activation of the muscle metaboreflex, are also activated by the increased muscle blood flow at the onset of exercise [27]. Thus, it was tempting to speculate that the limitation of blood and oxygen supply to exercising muscle in COPDs and overweight subjects may alter the muscle sensory pathways, delaying the response to unloaded pedalling. It is well known that the exercise pressor reflex induces cardiovascular adjustments to exercise via increases in sympathetic nerve activity and by withdrawal of parasympathetic nerve activity [28] and the beta-adrenergic and parasympathetic control of HR may be different between our groups.

We reported a significant elevation of the plateau minute ventilation increase during unloaded cycling in all patients with overweight compared to controls, whatever their baseline PaO_2_ level. Their ventilatory response differed from controls through a prevailing increase in respiratory frequency. This was mostly present in the hypoxemic group which had also the highest overweight. It is documented that increase during incremental cycling exercise was higher in individuals with upper body adiposity than in lean subjects [29]. This effect was also present in experiments in healthy lean subjects reproducing the overweight-related limitation of chest wall mechanics [7, 8]. The restriction of lung volumes in our overweight subjects was not correlated with the magnitude of their accentuated ventilatory response to unloaded cycling, indicating that spirometric indices are not reliable predictive indicators of loaded breathing at work.

We noted an elevated plateau HR response in our hypoxemic subjects (COPDs and individuals with overweight). This observation is poorly documented in the literature. In COPDs with partial pressure of oxygen in arterial blood around 65 mmHg, Schrijen and coworkers [30] have reported a larger increase in systemic arterial pressure during constant load supine bicycling from loadless to 30 W and they attributed the enhanced circulatory response to the vasoconstrictor effect of hypoxia. Unfortunately, they did not simultaneously measure cardiac variables. Experiments in healthy subjects exposed to normobaric hypoxia showed an accentuated heart rate and activation of muscle sympathetic nerve activity in response to rhythmic handgrip exercise [31, 32]. We supposed that the enhanced heart rate increase during unloaded cycling in our hypoxemic subjects (patients with COPD or overweight) may result from the potentiation by hypoxemia of the exercise-induced sympathetic neural response.

## 5. Conclusion

The present data indicate a predictive value of the ventilatory but not heart rate response to unloaded cycling on performances at higher workload. The relationship between VE increase during unloaded cycling and VE changes measured at both ventilatory threshold and maximal exercise power may have some interest to predict the ventilatory performance at work not only in healthy individuals but also in COPD and overweight subjects included in an exercise rehabilitation program.

## Figures and Tables

**Table 1:** Morphological characteristics, physiological data at rest, and oxygen uptake at the different epochs of exercise. Asterisks denote significant variations compared to controls (^*^ *P* < 0.05; ^**^ *P* < 0.01; and ^***^ *P* < 0.001). For pulmonary function, values in brackets are the percentage of predicted normal data.

	Controls	COPDs	Overweight subjects	
			Hypoxemic	Normoxemic
Number	18	14	12	19
Age, y	49 ± 6	54 ± 4	55 ± 2	53 ± 3
Body mass index, kg·m^−2^	23 ± 1.0	24 ± 1.5	31 ± 2.0^**^	29.4 ± 2.2^*^
Forced vital capacity (FVC), lBTPS (% predicted)	4.54 ± 0.39 (101%)	3.56 ± 0.20 (68%)	3.79 ± 0.36^*^ (70%)	4.12 ± 0.50 (97%)
Expiratory reserve volume (ERV), lBTPS	1.37 ± 0.20	1.03 ± 0.15	0.72 ± 0.35^*^	0.86 ± 0.25
Total lung capacity (TLC), lBTPS (% predicted)	6.72 ± 0.39 (102%)	6.45 ± 0.34 (100%)	5.81 ± 0.36^*^ (75%)	5.92 ± 0.31^*^ (78%)
Forced expiratory volume in 1 s (FEV1), lBTPS (% predicted)	3.89 ± 0.33 (96%)	2.38 ± 0.25^**^ (60%)	3.18 ± 0.23 (81%)	3.27 ± 0.18 (86%)
FEV1/FVC, %	86	67	84	79
PaO_2_	85	67^***^	72^*^	85
SpO_2_, %	99 ± 0.8	95 ± 0.6	96 ± 0.5	99 ± 0.7
Heart rate, min^−1^	72 ± 3	81 ± 5	69 ± 4	77 ± 3
Minute ventilation (VE), lBTPS·min^−1^	11	11	10	13
Respiratory frequency (fR), min^−1^	16	18	15	17
Oxygen uptake (VO_2_), mlSTPD^−1^·kg^−1^ VO_2_ rest	4.9 ± 0.2	4.5 ± 0.2	4.2 ± 0.2	4.3 ± 0.3
VO_2_ end unloaded cycling	8.1 ± 0.4	8.2 ± 0.6	7.3 ± 0.3	7.4 ± 0.4
VO_2_ threshold	21.2 ± 1.0	16.2 ± 0.9^**^	15.2 ± 1.4^**^	18.4 ± 0.8^*^

## References

[1] Cote C. G., Pinto-Plata V., Kasprzyk K., Dordelly L. J., Celli B. R. (2007). The 6-min walk distance, peak oxygen uptake, and mortality in COPD. *Chest*.

[2] Ross R. M., Murthy J. N., Wollak I. D., Jackson A. S. (2010). The six minute walk test accurately estimates mean peak oxygen uptake. *BMC Pulmonary Medicine*.

[3] Oga T., Nishimura K., Tsukino M., Sato S., Hajiro T., Mishima M. (2005). Exercise capacity deterioration in patients with COPD: longitudinal evaluation over 5 years. *Chest*.

[4] Sietsema K. (2001). Cardiovascular limitations in chronic pulmonary disease. *Medicine and Science in Sports and Exercise*.

[5] Tzani P., Aiello M., Elia D. (2011). Dynamic hyperinflation is associated with a poor cardiovascular response to exercise in COPD patients. *Respiratory Research*.

[6] Negrão C. E., Trombetta I. C., Batalha L. T. (2001). Muscle metaboreflex control is diminished in normotensive obese women. *The American Journal of Physiology—Heart and Circulatory Physiology*.

[7] Parameswaran K., Todd D. C., Soth M. (2006). Altered respiratory physiology in obesity. *Canadian Respiratory Journal*.

[8] Trombetta I. C., Batalha L. T., Rondon M. U. P. B. (2003). Weight loss improves neurovascular and muscle metaboreflex control in obesity. *The American Journal of Physiology—Heart and Circulatory Physiology*.

[9] Lorenzo S., Babb T. G. (2012). Quantification of cardiorespiratory fitness in healthy nonobese and obese men and women. *Chest*.

[10] Lorenzo S., Babb T. G. (2013). Ventilatory responses at peak exercise in endurance-trained obese adults. *Chest*.

[11] Zattara-Hartmann M. C., Jammes Y. (1996). Acute hypoxemia depresses the cardiorespiratory response during phase I constant load exercise and unloaded cycling. *Archives of Physiology and Biochemistry*.

[12] Keslacy S., Matecki S., Carra J. (2005). Effect of inspiratory threshold loading on ventilatory kinetics during constant-load exercise. *American Journal of Physiology—Regulatory Integrative and Comparative Physiology*.

[13] Wang L.-Y., Cerny F. J. (2004). Ventilatory response to exercise in simulated obesity by chest loading. *Medicine and Science in Sports and Exercise*.

[14] Celli B. R., MacNee W., Agusti A. (2004). Standards for the diagnosis and care of patients with chronic obstructive pulmonary disease (COPD) and asthma. *European Respiratory Journal*.

[15] Bechbache R. R., Duffin J. (1977). The entrainment of breathing frequency by exercise rhythm. *Journal of Physiology*.

[16] Paterson D. J., Wood G. A., Morton A. R., Henstridge J. D. (1986). The entrainment of ventilation frequency to exercise rhythm. *European Journal of Applied Physiology and Occupational Physiology*.

[17] Quanjer P. H. (1983). Standardized lung function testing. *Bulletin Européen de Physiopathologie Respiratoire*.

[18] Siggaard-Andersen O., Siggaard-Andersen M. (1990). The oxygen status algorithm: a computer program for calculating and displaying pH and blood gas data. *Scandinavian Journal of Clinical and Laboratory Investigation*.

[19] Davis J. A., Frank M. H., Whipp B. J., Wasserman K. (1979). Anaerobic threshold alterations caused by endurance training in middle-aged men. *Journal of Applied Physiology Respiratory Environmental and Exercise Physiology*.

[20] Maillard D., Gautier H. (1981). Gas exchange during bicycle exercises preceded or not by loadless pedalling in female and male subjects. *Respiration Physiology*.

[21] Porszasz J., Casaburi R., Somfay A., Woodhouse L. J., Whipp B. J. (2003). A treadmill ramp protocol using simultaneous changes in speed and grade. *Medicine and Science in Sports and Exercise*.

[22] Thys H., Willems P. A., Saels P. (1996). Energy cost, mechanical work and muscular efficiency in swing-through gait with elbow crutches. *Journal of Biomechanics*.

[23] Jammes Y., Mathiot M. J., Roll J. P. (1981). Ventilatory responses to muscular vibrations in healthy humans. *Journal of Applied Physiology Respiratory Environmental and Exercise Physiology*.

[24] Jammes Y., Askanazi J., Weissman C., Milic-Emili J. (1984). Ventilatory effects of biceps vibration during leg exercise in healthy humans. *Clinical Physiology*.

[25] Jammes Y., Guillot C., Prefaut C., Grimaud C. (1976). Relationships between eupnoeic pattern of breathing and ventilatory control in man. II. Early response to transient hypercapnia. *Archives Internationales de Physiologie et de Biochimie*.

[26] Simon M., LeBlanc P., Jobin J., Desmeules M., Sullivan M. J., Maltais F. (2001). Limitation of lower limb VO_2_ during cycling exercise in COPD patients. *Journal of Applied Physiology*.

[27] Haouzi P. (2006). Theories on the nature of the coupling between ventilation and gas exchange during exercise. *Respiratory Physiology and Neurobiology*.

[28] Smith S. A., Mitchell J. H., Garry M. G. (2006). The mammalian exercise pressor reflex in health and disease. *Experimental Physiology*.

[29] Li J., Li S., Feuers R. J., Buffington C. K., Cowan G. S. M. (2001). Influence of body fat distribution on oxygen uptake and pulmonary performance in morbidly obese females during exercise. *Respirology*.

[30] Schrijen F., Mohan-Kumar T., Polu J. M. (1991). Circulatory response to repeated exercise in patients with chronic lung disease. *Respiration*.

[31] Hanada A., Sander M., González-Alonso J. (2003). Human skeletal muscle sympathetic nerve activity, heart rate and limb haemodynamics with reduced blood oxygenation and exercise. *The Journal of Physiology*.

[32] Seals D. R., Johnson D. G., Fregosi R. F. (1991). Hypoxia potentiates exercise-induced sympathetic neural activation in humans. *Journal of Applied Physiology*.

